# Remarks on Fistula in Ano*Read before Georgia Medical Association, April, 1897.

**Published:** 1897-07

**Authors:** Wm. S. Goldsmith

**Affiliations:** Atlanta, Ga., Lecturer on Genito-Urinary and Rectal Diseases, Atlanta Medical College


### REMARKS ON FISTULA IN ANO*

BY WM. S. GOLDSMITH, M.D., ATLANTA, GA.,

Lecturer on Genito-Urinary and Rectal Diseases, Atlanta Medical College.

The relative frequency of fistula in ano as compared with other rectal affections, induces me to a consideration of this interesting, though perhaps well-worn subject. Some differences of opinion exist among authors as to the frequency of fistula. Allingham states that in a collection of 4,000 consecutive rectal cases in the out-patient department of St. Mark's Hospital, 1,057 were fistula in ano, 196 abscesses, 151 of which subsequently became fistula, and that for a period of several years, two-thirds of the cases operated on in this hospital were for fistula.

Matthews, on the other hand, states that while the margin is small, internal hemorrhoids have occurred more frequently in his practice. Omitting the simpler forms of rectal affections, such as fissure and ulcer, and accepting fistula and internal hemorrhoids as the most common, I am inclined to place the former at the head of the list. This is my experience in private practice, and that also of the cases presented at one of the largest outdoor surgical clinics in the South. Fistula, therefore, occupying such a prominent position among the more common and grave surgical diseases, I ask your indulgence that I may briefly bring out some points underlying the principles of treatment.

*Read before Georgia Medical Association, April, 1897.

In common with all other practitioners, I am inclined to a certain degree of partiality in the use of the expression, "conservative surgery," but in the discussion of this affection I shall for once endeavor to escape its fascinative euphony. Individual experience has amply demonstrated that only the most radical and thorough technique is productive of the permanent results so anxiously sought, and any deviation from this rule is most likely to redound to our discredit. I trust that my attitude in laying such particular stress on this point will not be charged to exaggerated presumption, as I am sure that all of us can recall an instance in our surgical work where a poor result is safely attributed to some negligence in technique or undue trepidation in method.

I feel no hesitancy in declaring that the incision of all fistulous tracts and sinuses, and the complete section of the sphincter muscles, with the knife, is the only method by which we can, with considerable certainty, assure the patient that the operation will result in a rapid and successful cure. The methods of ligation and chemical irritation will not, therefore, enter into the discussion. The fallacious methods advocated by the older writers cannot be other than productive of most unsuccessful and humiliating results. For instance, Hamilton, in his Principles and Practice of Surgery, says: "The probe or somewhat flexible groove director, being now thrust into the rectum and brought out at the anus, the operation is completed by dividing the intermediate tissues. Having cut the sphincter, it only remains to lay a small piece of lint between the margins of the wound and place the patient in bed."

Dr. Matthews, in his book on Diseases of the Rectum, corrects this proposition in the following admirable manner: "To illustrate how erroneous the advice is, allow me to cite a case: If an abscess in the ischio-rectal fossa has left a sinus which runs directly into the bowel, and from this a branch fistula runs out into the perineum, and another diverges from the main channel into the buttock, no such operation as is described by Hamilton would effect a cure. It is the smallest part of the operation to lay open the tissues which lie over the main sinus. How often it is that the surgeon is disappointed in the wounds refusing to heal after an operation for fistula, and an investigation reveals that it is due to a small sinus or pocket that has been overlooked! I am sure after a long experience in dealing with this operation, that in the majority of cases operated upon, if a single sinus is left, a good result will not be obtained. In other words, the inflammation excited will not be sufficient to eradicate the branch fistula. The flaps or thin edges of the wound alone, if left, would prevent good union."

I have yet to see a wound made in the area usually involved fail to promptly and satisfactorily granulate, provided it has had the benefit of a thorough eradication of all morbid tissues. To further elucidate this point, suppose all branches and pockets have been opened, should the operation be pronounced completed? Decidedly no. And just here my friend, Dr. Matthews executes a procedure that is rather tedious, and, I think, quite unnecessary. He advises the excision of the whole bottom of the wound, using pinch forceps and curved scissors, and states that he is not satisfied with simply scraping out the sinuses. I think the careful use of the Volkman spoon, together with the application of peroxide of hydrogen, in the manner to be described, is far preferable. After incising the main tract and all sinuses, satisfying myself that the superior extremity of the original tract is reached, and making a rather prolonged search for pockets and hidden branches, I trim away the overhanging edges of skin along the entire cut area, paying particular attention to the edges and vicinity of the original external apertures.

This is followed by a thorough curettage of the bottom of the sinus, and the removal, as far as possible, of every particle of the so-called pyogenic membrane. The wound is irrigated and sponged as dry as circumstances will permit. I now use peroxide of hydrogen, full strength, applied in the form of a spray by an ordinary hand atomizer. It is pleasing to observe the excellent effect this valuable agent has on the débris unavoidably retained in the wound. The hydrogen used in this manner penetrates into every minute pocket and cuts out particles of tissue that it is impossible to remove, except by the total excision of the entire lining membrane. Having decided acid reaction, it is most valuable in checking the otherwise profuse oozing accompanying the simple curetting or excision. The splendid results attained by the use of peroxide of hydrogen in the manner described, is obtainable in other lines of work where the same action is desired, and has been used by Dr. Westmoreland and myself most satisfactorily in the treatment of chronic osteo-myelitis and all varieties of indolent ulcers.

I now wish to invade the precincts of conservative surgery: Where there are two or more internal openings into the bowel, widely divergent, sever only one at a sitting. Of course if the openings are in close proximity, the exercise of some ingenuity may be practiced and virtually one cut through the muscle will suffice; but if any doubt exists as to your ability to accomplish this, err on the right side, if erring it is. A recent case presented four distinct tracts leading to one internal opening. These were all apparently primary sinuses, as each had from two to four branches. My relief can be imagined when I found that only one internal opening existed, necessitating a single section of the muscle. It is unpleasant to relate that operations have been done where even a single section of the muscle was avoided. It is apparent to all the degree of absurdity and mutilating barbarity abounding in such work. What line of reasoning is pursued to induce such a stupid procedure, is beyond my comprehension. Another interesting and most valuable point in connection with the technique, is the recognition of the influence a contracted sphincter muscle plays in the actual performance of the operation and the tremendous effect involved in the repair of the wound. We know that any irritation in the vicinity of the muscles will cause a contracted condition which is continued so long as the irritant is present, and is productive of so many reflex phenomena, aside from the discomfort accompanying defecation. If this muscle is not stretched, this spasm remaining unrelaxed, mere section of the muscle not only does not abolish, but vastly increases the irritation, with accompanying increase in spasm. Consider the effect of this condition on the effort of nature to bring together the ends of this muscle. It is like a balking horse. The more we whip, the more unreasonable it becomes. On the other hand, suppose the muscle is thoroughly stretched, carefully and slowly, and with the intention of relaxing each individual fiber. The divulsion temporarily paralyzes the muscle, and for a few hours has absolute rest and quiet. The nerve ends gain their freedom and the pain and reflexes are consequently obliterated. The muscle having had a good rest, does not now resent the process of repair and joins in with increased vigor toward the accomplishment of a rapid recovery. This divulsion of the sphincter muscles is also especially indicated in operations for hemorrhoids, and in fact all operations in and about the rectum where physiological recuperation of functional activity is so essential to a cure.

The actual cautery is a most valuable adjuvant in the treatment of old sinuses already operated upon, and through some error of management or the run-down condition of the patient, fail to respond to our efforts to heal. There is no agent, in my judgment, so material in stimulating granulation to new and active growth. Patients are usually greatly delibitated as the result of old fistula and sometimes make exceedingly slow progress towards regaining their wonted health. I have prescribed, with happy effects, a mercurial in the form of one-eighth grain to one-fourth grain pretoiodide mercury, with extract gentian. This combination is not only an excellent tonic, but is very beneficial in toning up the liver, thereby rendering the use of laxatives and enematas wholly unnecessary. As an absorbent it is also very efficient, as often there are masses of indurated tissue left which can only be removed by this method.

The question as to the influence of pulmonary tuberculosis on radical operations for fistula in ano has been so widely discussed that only slight reference need be made in this connection. I believe the consensus of opinion among the authorities is that as a general proposition, the presence of phthisis should have absolutely no weight with us in deciding upon and performing such operations as we see fit. Personally, I adhere rigidly to this view, and have yet to see a satisfactory reason why I should not continue to do so. I have seen several cases where there were decided symptoms of phthisis, and from such later information as I could obtain, nothing but the happiest results were secured. Our only guide is the physical condition of the patient. If he is strong enough to take the anesthetic and resist the shock attendant upon an operation of such magnitude, we should not be deterred from rendering such operative assistance as our judgment dictates.

*27-28 Grant Building.*

